# Dr. Arthur Gamgee

**Published:** 1909-04-03

**Authors:** 


### OBITUARY.

Dr. Arthur Gamgee, M.D., F.R.S., F.R.C.P., of 66 Harley Street, died in Paris on Monday in his 68th year. He was Emeritus Professor of Physiology in the Victoria University, and had been assistant physician to St. George's Hospital, London, where he was also lecturer on materia medica in the Medical School. He became a Fellow of the Royal Society at a very early age as the reward of special research work in connection with some of the most abstruse problems of physiological chemistry; and he held, from time to time, a number of important offices in the University of Edinburgh, in Owens College, and in the Royal Institution of Great Britain. Much of his work was conducted in the physiological laboratories of Germany, France, and Switzerland. His "Text-book of Physiological Chemistry," in which much original research was incorporated, has been translated into French and German. Dr. Gamgee was a fine classical scholar and a good linguist. His unexpected death was due to post-influenzal pneumonia. His loss will be felt by a wide circle of scientific men in this country and abroad.

THE HOSPITAL April 3, 1909.

